# HtPIP: High-throughput phage isolation platform increases diversity and reduces isolation time using multiple bacteria

**DOI:** 10.3389/fmicb.2026.1845440

**Published:** 2026-06-18

**Authors:** Ben Diaz, Tessa House, Meghana Padala, Joseph S. Schoeniger, Catherine M. Mageeney

**Affiliations:** 1 Department of Bioengineering and Biotechnology, Sandia National Laboratories, Livermore, CA, United States; 2 Biological, Radiation, and Signature Science, Technology, and Engineering Center, Sandia National Laboratories, Livermore, CA, United States

**Keywords:** high-throughput, phage discovery, phage diversity, phage genome, phages

## Abstract

Bacteriophages are ubiquitous in nature, but relatively few have been isolated and characterized compared to the number of bacterial strains. Phage biotechnology applications benefit from a diverse library of isolated phages to kill or transfer genetic material to a bacterium of interest. However, scaling up phage discovery for diverse bacterial hosts can be time-consuming and costly. We developed an approach to capture novel phages for multiple bacterial strains in parallel from an environmental sample using commercially available 0.2-μM filter plates. Using this High-throughput Phage Isolation Platform (HtPIP), 12 novel phages were isolated spanning 9 diverse bacterial host genera. Eleven of the isolated phages define new phage species, with nine also defining new genera. The HtPIP was used to discover both DNA and RNA phages, including a *Tectiviridae* infecting *Pseudomonas putida* mt-2 and a *Leviviricetes* infecting a *Microbacterium* isolate, which represents the first cultured RNA phage infecting a host outside of Proteobacteria. Using a metagenomic approach, we demonstrate that the HtPIP captures a higher proportion of novel phages compared to traditional low-throughput methods.

## 1 Introduction

Bacteriophages (phages) are viruses that infect bacteria. They are found in almost every environmental niche on Earth that supports bacterial life, including wastewater (Abe et al., 2014), soil (Graham et al., 2024), the ocean (Eppley et al., 2022), and mammalian microbiomes (Shkoporov et al., 2022). The majority of the phages have access to two lifecycles, which allow the bacteria/phage relationship to be predatory during the lytic lifecycle and mutualistic during the lysogenic lifecycle (Mageeney et al., 2020; Dedrick et al., 2017; Bondy-Denomy et al., 2016). Additionally, phages can carry novel auxiliary metabolic genes to enhance host metabolism (Wu et al., 2022) or genes that enhance host pathogenesis or predator resistance. The diversity of phages in the biosphere allows for a wide range of applications in biotechnology as gene delivery vehicles (Karimi et al., 2016), sources of new synthetic and molecular components (Lammens et al., 2020; Lemire et al., 2018), as biocontrol agents to prevent plant disease (Nakayinga et al., 2021; Huss and Raman, 2020), agents to combat biofilms (Figueiredo et al., 2021), and as therapeutics for antibiotic-resistant infections in humans and animals (Hatfull et al., 2022; Gordillo Altamirano and Barr, 2019).

The majority of bacterial strains lack isolated phages for phage-based biotechnology applications. Many applications typically require a cocktail comprised of several complementary phages (Hatfull et al., 2022) to prevent prompt phage resistance, further widening this availability gap. It follows that for many phage-based applications to be successful, rapid discovery methods or a phage bank with numerous diverse phages for each bacterial host is required. Traditional phage isolation is, however, low-throughput and costly for multiple hosts, and thus does not always yield the quantity or diversity of phages needed for a phage bank (Weber-Dąbrowska et al., 2016; Hyman, 2019).

A typical process for isolating phages from the environment is to add the environmental sample (soil, water, or sewage) to a culture of a single bacterial host of interest, incubate, and then recover the phage particles and remove the bacteria (typically by filtration). The filtrate is then tested for the presence of phages for the bacteria of interest (Hyman, 2019) typically by plaque assay. While methods for higher-throughput phage discovery have been developed in recent years by scaling this process for a 96-well plate (Olsen et al., 2020) they can remain time-consuming and costly due to multiple manual filtration steps. Inspired by the iChip, which enables culturing of previously uncultivable bacteria by growing them within a compartment enclosed by semipermeable membranes within an environmental sample (Nichols et al., 2010), we sought to design a high-throughput method that captures a higher diversity of native phages by allowing low-abundance phages to bloom alongside the native bacteria living in the environmental samples (Wu et al., 2023). In addition, we aimed to reduce the labor-intensive and potentially hazardous steps of centrifugation and filtration of environmental samples (Göller et al., 2020; Trubl et al., 2019).

We developed and tested the feasibility of a High-throughput Phage Isolation Platform (HtPIP). We sought to establish a robust method with commercially available materials to ensure reproducibility and reduce phage isolation time for multiple bacterial strains concurrently. Using HtPIP, twelve novel phages were isolated infecting nine bacterial hosts. These include diverse morphotypes (*Siphoviridae*, *Myoviridae*, *Tectiviridae*, and *Leviviridae*) and numerous new phage genera and species. Furthermore, the first *Leviviricetes* infecting a Gram-positive host (*Microbacterium*) was isolated, demonstrating the utility of HtPIP to capture both DNA and RNA viruses. We then explored the potential diversity of the metavirome captured from wastewater influent incubated with *Escherichia coli* and found that a higher proportion of novel and diverse phages are captured with this HtPIP vs. traditional low-throughput methods.

## 2 Materials and methods

### 2.1 Bacterial cultures

Bacteria were grown overnight, shaking at 200 rpm at 20 °C. A temperature of 20 °C was chosen to allow all bacteria to grow and be infected by phages, as *Pseudomonas putida* has been shown to defend against phage infection at 37 °C (Brauer et al., 2024). The following bacteria were grown in Lysogeny Broth (LB) media (Sigma-Aldrich): *E. coli* MG1655*, P. putida* mt-2 (ATCC 33015)*, P. putida* S12, *Bacillus subtilis* NCIB 3610, and *Burkholderia cenocepacia* K56-2 (obtained from BEI NR-20535). The following bacteria were grown in R2A media (Teknova): Var*iovorax* sp. SCN*, Variovorax* sp. OAS795, *Rhodococcus rhodochrous* 372 (ATCC 13808), *Rhodococcus qingshengii* S10, *Rhodococcus* sp. MSC1_016, and *Sphingopyxis fribergensis* MSC1_008. *Microbacterium* sp. CM01 was grown in Difco International Streptomyces Project (ISP) media [Becton Dickinson (BD)]. All cultures were supplemented with 1 mM CaCl_2_ (Sigma-Aldrich).

The bacterial host *Microbacterium* sp. CM01 was not previously sequenced. Genomic DNA was prepared from *Microbacterium* sp. CM01 by pelleting 10 mL of culture, washing in 1 mL phosphate-buffered saline (PBS), then resuspending in 0.5 mL Zymo DNA/RNA Shield. The preserved cell pellets were sequenced at Plasmidsaurus using a Hybrid Illumina and Oxford Nanopore approach at 100 × final coverage. The resultant genome aligned at 99.32% ANI with the genus *Microbacterium* against a custom database in Plasmidsaurus (GTDB rs226) using sourmash (v5) (Irber et al., 2024). CheckM (v1.2.2) (Parks et al., 2015) estimated the genome completeness at 99.49% with 0.0% contamination.

### 2.2 HtPIP optimization

Lambda was diluted in phage buffer to 1 × 10^6^ PFU/mL in the sterilized sample reservoir, and the filter plate was loaded with *E. coli* MG1655 and multiple negative controls (phage buffer, LB broth, top agar). The loaded plate was placed on top of the Lambda suspension, shaken for 1 h at 50 rpm at 20 °C (room temperature), and incubated overnight at 37 °C. The next day, liquid wells were pooled and filtered by hand via a 0.2 μM syringe filter, while top agar cultures were mixed with 200 μL of phage buffer, pooled, and then filtered by hand via a 0.2 μM syringe filter.

### 2.3 Environmental sample collection

Samples were collected from the sites listed in Table 1. Untreated wastewater influent samples were collected with permission from the Livermore Water Reclamation Plant in Livermore, CA, USA. A 250 mL liquid sample was collected from wastewater and pond sources, while approximately 100 mL volume of solid samples was collected from each solid source. For topsoil and agricultural soil, we collected 100 mL (~150 g), and for less-dense soils such as compost, we collected 200 mL. Volume was prioritized over mass since the soil samples were highly variable in density and composition.

**Table 1:** Sampling sites used in this study.

Sample	GPS coordinates	Sample state
Wastewater influent 1–3	37.69073324466393, −121.80667889554306	Liquid
Tomato rhizosphere	37.68343382977756, −121.74488605243637	Solid
Compost - backyard	37.93132569198107, −121.7123907950711	Solid
Topsoil	Purchased from a home improvement retailer	Solid
Compost - community center	37.677616812367795, −121.73652562520904	Solid
Washington State University field site in Prosser, WA	46.252480744413305, −119.73798155092547	Solid

Samples were collected from a diverse set of sites in the San Francisco East Bay, USA, and an agricultural field site in Washington State, USA. The sample name, global positioning system (GPS) coordinates, and the sample state (liquid/solid) are listed.

### 2.4 Traditional phage isolation

Traditional enrichment filtrates were processed and filtered the same day HtPIP was started. Liquid environmental samples were filtered through a 0.2-μM filter [Millipore Express Polyethersulfone (PES) Membrane]. Sodium chloride-magnesium sulfate (SM) buffer without gelatin [150 mM NaCl (Sigma-Aldrich), 40 mM Tris-Cl (pH 7.4) (Teknova), and 10 mM MgSO_4_ (Sigma-Aldrich)] was added to solid samples to make a slurry, inverted several times gently, and centrifuged for 30 min at 5,000 relative centrifugal force (RCF). The supernatant was filtered through a 0.2-μM filter. Enrichment cultures were set up with 1 mL of filtrate, 4 mL of fresh LB liquid supplemented with 1 mM CaCl_2_, and 50–1,000 μL of an overnight bacterial culture. Overnight cultures of faster growing bacteria (*E. coli*, *P. putida,* and *B. subtilis*) were added at lower ratios (50 μL: 1:80 dilution). In comparison, all other bacteria used were added at higher ratios (1,000 μL: 1:4 dilution). These mixtures were grown for ~68 h at 20 °C, shaken at 200 rpm, then filtered again through a 0.2-μM filter. Double-agar overlays were used to determine if phages were present for hosts of interest by mixing 250 μL enrichment filtrate, 50–1,000 μL of overnight bacterial culture, and 3 mL of LB top agar (0.5% agar, 1 mM CaCl_2_) and plated onto LB Agar plates supplemented with 1 mM CaCl_2_.

### 2.5 HtPIP phage isolation

Freshly diluted bacterial cultures at the same ratio described above (1:80–1:4) were loaded into a MultiScreen-GV Filter Plate (Millipore Sigma, MAGVS2210), with the underdrain removed. Inside a pre-autoclaved polypropylene tray with lid (SP Bel-Art/VWR 62663–222), a 100–200 mL slurry of solid substrate (soil or compost) and SM buffer, or 100 mL of environmental sample (unfiltered wastewater) was added before placing the filter plate loaded with bacterial cultures on top and sealing the lid with parafilm. HtPIP plates were incubated at 20 °C, 100 rpm for 68 h. A separate plate was used to filter the subsequent enrichment cultures with the underdrain intact. Liquid samples were processed by transferring 150 μL from each well to a new filter plate placed on top of a 96-well plate (Nunc 96-Well Polystyrene Round Bottom Microwell Plates – catalog number: 268200) and centrifuged for 10 min at 2,000 RCF. Semi-solid samples (0.5% agar with growth media [i.e., LB, ISP, and R2A], melted and kept molten at 55 °C) wells were processed by adding 100 μL of phage buffer to each well, pipetting up and down several times slowly, then transferred to the clean filter plate. If the sample did not filter, centrifugation was repeated up to 2 more times.

### 2.6 Phage purification of isolated phages

In samples where plaques or lawn clearing were observed, 2 individual plaques were picked, resuspended in 100 μL SM buffer, and diluted to 10^−5^. These dilutions were mixed with fresh overnight bacterial culture diluted in each host’s respective molten top agar media (LB, R2A, or ISP). This process was repeated for a total of three purifications, wherein lawns were flooded with 8 mL of SM buffer, scraped, briefly centrifuged (500 RCF for 5 min), and the supernatant was filtered through a 0.2 μM filter. Genomic DNA was extracted from these purified lysates using Norgen Phage DNA Isolation kits without modification. Libraries were prepped with the Illumina DNA Prep Tagmentation kit and Illumina Unique Dual Indexes.

For phages where the Norgen kit did not yield high-quality DNA (>20 ng/μL and A260/230 > 2, and A260/280 > 1.8), lysates were processed by SeqCoast Genomics (Portsmouth, NH, USA), where samples were lysed using MagMAX Microbiome bead beating tubes. DNA was extracted using the Qiagen DNeasy 96 PowerSoil Pro QIAcube HT Kit (as listed in Supplementary Table S1).

*Microbacterium* phage Later genomic RNA was extracted using the Monarch Mag Viral RNA/DNA extraction kit (New England Biosciences). Libraries were prepped using the Illumina Stranded Total RNA Prep with Ribo-Zero Plus kit without modifications and sequenced on an Illumina NextSeq 2000.

All DNA libraries were sequenced using Illumina sequencing platforms (see Supplementary Table S1 for platform details).

### 2.7 Phage Later characterization

To determine the host range of *Microbacterium* phage Later, 10 μL of purified phage lysate was directly spotted, without dilution, onto double agar overlays containing *R. rhodochrous* 372 (ATCC 13808), *R. qingshengii* S10, and *Rhodococcus* sp. MSC1_016, *Streptomyces* sp. MSC1_001, *Streptomyces* sp. MSC1_017, *Streptomyces venezuelae* ATCC 10712, and *E. coli* MFDpir. The *Rhodococcus* strains were grown at 30°C in R2A media supplemented with 1 mM CaCl_2_, the *Streptomyces* strains were grown at 25°C in ISP media supplemented with 1 mM CaCl_2_, and *E. coli* MFDPir was grown in LB supplemented with 0.3 mM diaminopimelic acid and 1 mM CaCl_2_ at 37°C. To determine whether calcium was necessary to propagate *Microbacterium* phage Later, purified lysate was diluted in R2A media and spotted on double-agar overlays of *Microbacterium* sp. CM01 with and without 1 mM CaCl_2_ supplementation. To determine the infection temperature range of *Microbacterium* phage Later, spotted plates with and without calcium were incubated separately at a range of temperatures (20°C, 25°C, 30°C, and 37°C). To determine if *Microbacterium* phage Later was able to infect in planktonic/liquid conditions in addition to biofilm/semisolid conditions, 200 μl of a 1/200 dilution of *Microbacterium* sp. CM01 was added to 96-well plates (Corning flat-bottom untreated sterile polystyrene), along with 2 μl of phage Later lysate diluted in R2A media, and the optical density (OD) was monitored on a Tecan Spark.

### 2.8 Phage metavirome collection

Metaviromes were generated from traditional and HtPIP methods. The same overnight culture of *E. coli* MG1655 was diluted to the same concentration in liquid (50 μL overnight culture/mL sterile LB supplemented with 1 mM CaCl_2_) and loaded onto a filter plate “HtPIP Liquid” or added to 1 mL LB top agar (“HtPIP Semi-Solid”), or aliquoted into a tube with 1 mL 0.2 μM filtered wastewater (“Traditional”). All HtPIP samples were sealed with Microseal “B” PCR Plate sealing film (Bio-Rad) and placed on top of 100 mL unfiltered wastewater. All samples were incubated at 20 °C, at 100 rpm for 68 h.

Enrichment cultures were filtered using a 0.2 μM syringe filter (“Traditional”) or by centrifugation (“HtPIP”), and then serially diluted, combined with freshly diluted LB top agar, and plated on LB Agar plates using the double-agar overlay technique. After overnight incubation at 20 °C, phage buffer was added to the plates, and the top agar was gently scraped. The top agar/phage buffer slurry was centrifuged for 10 min at 4,000 RCF, then filtered through 0.2 μM syringe filter.

DNA was extracted from 1 mL of each enrichment lysate using a phenol-chloroform extraction. Before extraction, Proteinase K (New England Biolabs) and 0.05% sodium dodecyl sulfate (SDS) (Sigma-Aldrich) were added, and the lysates were incubated at 55 °C with gentle shaking every 15 min. Next, an equal volume of phenol–chloroform–isoamyl alcohol mixture (25:24:1) was added to the samples and incubated at room temperature for 10 min. Samples were spun at 4 °C at 10,000 RCF for 10 min, and the aqueous phase was removed from the top. An equal volume of chloroform was added to the aqueous layer, which was incubated at room temperature for 10 min, centrifuged at 4 °C at 10,000 RCF for 5 min, and the aqueous phage was removed to a new tube. The chloroform extraction was repeated twice. The resulting aqueous extractions were then precipitated with PEG (final concentration 8 × %) and NaCl (final concentration 1 M). This was left to sit at room temperature for 30 min and then centrifuged at 10,000 RCF at room temperature for 30 min. The pellet was washed once in 100% ethanol and twice in 70% ethanol, both ice-cold, before drying for 15 min at 37 °C. The pellet was resuspended in deionized water and measured with QuBit before library preparation. Libraries were prepared with the Nextera XT kit and sequenced on a short-read Illumina platform at Genewiz (South Plainfield, NJ).

### 2.9 Isolate phage assembly and annotation

Raw sequencing reads for both metavirome experiments and isolated phages were processed with BBDuk (https://sourceforge.net/projects/bbmap/) with the following parameters specified (ktrim = r, k = 21, mink = 11, hdist = 1).

For isolating phage sequences, other than *Microbacterium* phage Later, trimmed high-quality reads were assembled using Spades (v3.9.0) (Bankevich et al., 2012) with the standard parameters for paired-end sequencing due to its high accuracy on isolated samples. The resulting spades assemblies were further filtered to remove small (<1,000 bp) and low-coverage assemblies (<1% maximum coverage). Final phage genomes were annotated using Prokka (v1.11) (Seemann, 2014) for open reading frame (ORF) calling and our DiMER pipeline (https://github.com/sandialabs/DiMER) for functional prediction. Resultant phage genome annotations were manually checked for any large gaps and to resolve translational frameshifts. tRNAscan-SE (v2.0.12) (Chan et al., 2021) was used to predict t(m)RNA genes in our phage genomes using the following additional parameters: -G -I. Phage genome statistics are listed in Supplementary Table S1. Phage taxonomy was predicted using the web-based version of taxMyPhage (Millard et al., 2025) and vContact2 (v0.9.19) (Bolduc et al., 2017) at KBase (Arkin et al., 2018). Phage genome maps were created with web-based Proksee (Grant et al., 2023). CheckV (Nayfach et al., 2021) (end_to_end, −t 5) was used to determine completeness. The nucleotide sequence for each phage was entered as the query in the core nucleotide database (core_nt) using the web-based Basic Local Alignment Search Tool for Nucleotides (BLASTN) to identify related phages. Web-based VIPTree (Nishimura et al., 2017) and VIRDIC (Moraru et al., 2020) were used to explore any evolutionary relationships with published phages.

Two clonal isolates of *Microbacterium* phage Later were sequenced. Raw reads from each were processed with BBDuk as described above. The trimmed high-quality reads were assembled using MEGAHIT (v1.2.9) (Li et al., 2015, 2016) with standard parameters for paired-end sequencing, given the sample’s sequencing depth. The resultant assemblies yielded multiple contigs (clone 1: 38 contigs and clone 2: 68 contigs) with a few larger than 1,000 bp (3 contigs and 9 contigs, respectively). BLASTN was used to determine if these contigs were bacterial or viral. Only one contig in each assembly aligned with a viral sequence [*Riboviria* sp. (OQ424403.1)]. These two contigs from the *Microbacterium* phage Later clonal isolates were aligned and showed 99.9% identity across 100% of the contig. The resultant contig was annotated as described above with Prokka. The predicted protein sequence for ORF3 was input into the web version of DeepTMHMM (Hallgren et al., 2022) to determine if orf3 was the lysis protein in *Microbacterium* phage Later.

### 2.10 Metavirome assembly and clustering

Trimmed high-quality reads from our metaviromic samples were assembled using MEGAHIT (v1.2.9) (Li et al., 2016; Li et al., 2015) with standard parameters. MEGAHIT was chosen for assembly of the metaviromic samples to align with current field protocols (Martínez-Puchol et al., 2024; Santos-Medellin et al., 2021) and to provide high-quality assemblies with lower computational resources required (van der Walt et al., 2017; Zhang et al., 2023). The MVP pipeline (Coclet et al., 2024) modules 00, 01, 02, 03, 04, and 05 were used to cluster the resulting viral contigs and obtain protein sequences for each viral contig as individual samples and across the entire dataset. VContact2 (Bin Jang et al., 2019) (version 0.11.3), with default settings, was used to cluster viral contigs. A graph was used to visualize clusters.

### 2.11 Transmission electron microscopy

Fresh high-titer lysates were sent for transmission electron microscopy (TEM) imaging on a Hitachi HT7800 by Tagide deCarvalho at the Keith R. Porter Imaging Facility, University of Maryland, Baltimore County.

## 3 Results

### 3.1 Development and optimization of HtPIP

Phage discovery using a semipermeable membrane plate arrayed with multiple bacterial strains was motivated by the goal of capturing a wider diversity of phages for a broader set of hosts more efficiently. We were inspired by the iChip system, which allows for the cultivation of diverse bacteria from the environment by allowing bacteria to exchange nutrients with their native habitat through a semipermeable membrane (Nichols et al., 2010). We reasoned diverse phages can bloom alongside bacteria in environmental samples, and that we could capture a wider variety of phages by tapping into this dynamic *in situ* pool. The parallel screening is aimed to increase the rate of success of finding a phage compared to traditional methods in two ways: (1) Given the inherent hit-or-miss during phage discovery for a specific host, screening diverse hosts in parallel increases the odds of capturing phages for some and (2) solid samples can be inhomogeneous, and phage may bloom at very low levels, so spatially distributed replicate wells containing the same host may increase the odds of capturing diverse phages for that host. Additionally, we aimed to remove the step of centrifuging and filtering environmental samples, which can be difficult and time-consuming, especially for soil samples (Göller et al., 2020).

**Figure 1:**
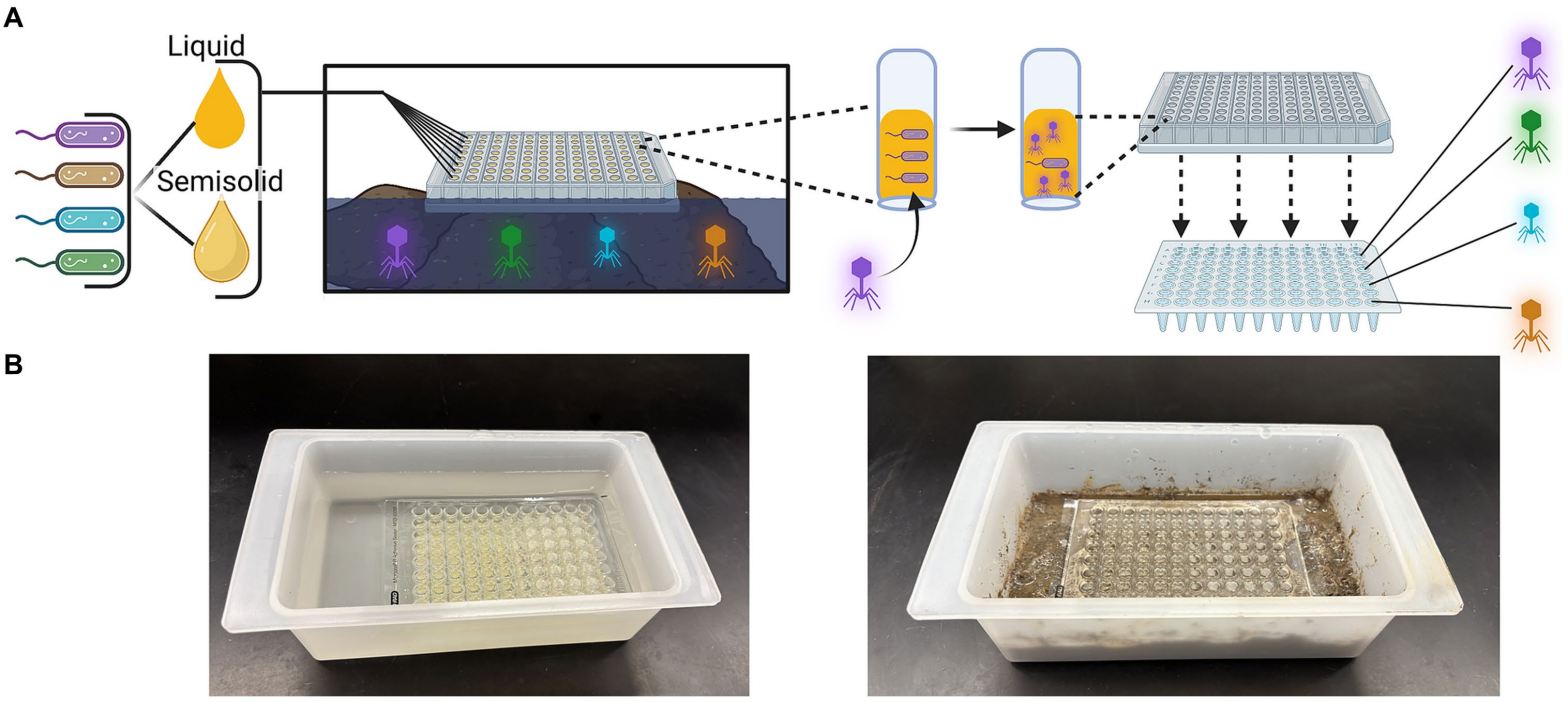
HtPIP setup and workflow. **(A)** Schematic of process. Bacteria are diluted into fresh liquid or semisolid media, then aliquoted into a semipermeable plate. The plate is placed on top of a soil slurry or liquid sample, allowing phages to percolate through the membrane, while containing bacteria. After incubation, the wells are filtered, and phages are recovered from the filtrate. **(B)** Photo of a plate incubating on top of a liquid (left) or soil slurry (right).

**Figure 2:**
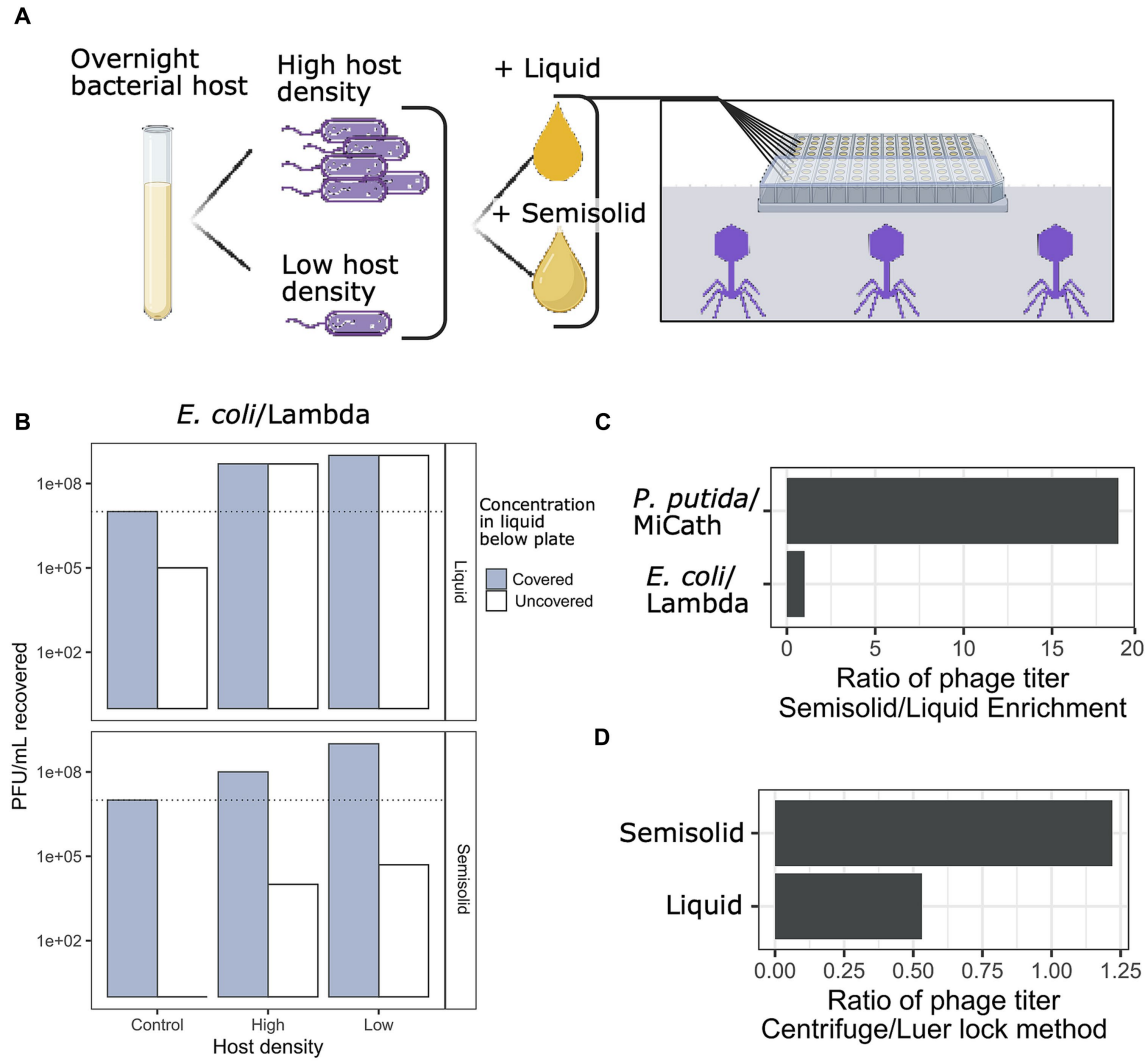
Optimization of phage isolation conditions using HtPIP. **(A)** Schematic of HtPIP conditions used for optimization with known phage concentrations in the reservoir below the plate. **(B)** Experiments with *Escherichia coli*/lambda combination, testing the effect of covering half of the plate with a PCR plate sealer (“Covered”/“Uncovered”), starting host density for enrichment (“High”/“Low” + control – only sterile media added to wells), and different enrichment media (“Liquid” – LB broth/ “Semisolid” - 0.5% LB agar). The dashed line represents the concentration of phages that were present in the liquid below the plate – a 1:1 ratio in the control means effectively all of the Lambda phage particles passed through the filter. **(C)** Testing the effect of enrichment media with a non-model host/virus combination (*Pseudomonas putida*/MiCath) on phage titer captured with the HtPIP (covered plates). Low starting host densities were used for both host/virus combinations. **(D)** Testing the effect of the filtering method after enrichment with *Pseudomonas putida*/MiCath with the HtPIP (covered plates). Centrifuge filtering saves time by processing the entire plate at once, whereas syringe filtering is a typical method for filtering each enrichment lysate by hand.

To ensure that this experimental system (Figure 1) was sufficient to meet these goals, several fundamental properties critical for phage discovery were tested, including phage transport through the 0.2-μM membrane, bacteria seeding density, media, and plate sealing. The model host-phage system (*E. coli* MG1655 and *coliphage* Lambda) was chosen to optimize HtPIP. An array of conditions was tested to determine which resulted in the highest yield of phage after enrichment: liquid vs. semi-solid (0.5% agar), host density: low (10^5^ colony-forming unit [CFU]/mL) vs. high (10^7^ CFU/mL), sealing: ambient airflow (hard plastic cover) vs. low airflow (Microseal adhesive B cover) (Figure 2A).

Lambda was shown to diffuse from the sample reservoir through the membrane to infect bacterial hosts as evidenced by ~100% phage recovery in the negative control (Figure 2B). A sealed top was necessary to capture phages when using semisolid media and significantly increased titers in liquid conditions (Figure 2B). It appeared that wells with lids left loose had less overall liquid, even in the media-only control wells, after incubating overnight. This may be a sign that the sealed plates prevent evaporation, or that they prevent the liquid equilibrating with the outer liquid level, which is slightly lower than the liquid level in the cultures growing inside the plates. Finally, there was a modest increase in phage titer at low starting host density on semisolid plates, and no change in phage recovered from liquid that depended on host density (Figure 2B).

To ensure the utility of our HtPIP plate for non-model bacteria and phages, *P. putida* S12 and phage MiCath (Jaryenneh et al., 2023) was used as a non-model phage-host system. MiCath was 18 × more concentrated when harvested from a semisolid enrichment compared to liquid enrichment (Figure 2C). Coliphage lambda did not show the same preference for semisolid enrichment, with an equal ratio of phages recovered from liquid and solid-covered plates (1×, Figure 2C). The effect of centrifuge filtering vs. manual syringe filtering was tested to ensure complete capture of phage particles, removal of bacteria, and the absence of sample-type bias. Both liquid and solid enrichment cultures could be filtered using both methods, and recovery yields were within a 2-fold range of each other (50 and 125% of hand filtering, respectively) (Figure 2D). This suggests that either method can be used to recover phages, but for liquid samples, a manual syringe outperforms centrifugation. These optimized conditions can be used to search for phages in a variety of environmental samples.

### 3.2 HtPIP enhances the discovery of novel phages for multiple bacterial hosts

Using the optimized HtPIP conditions (covered plate, low host density, centrifuge filtering, both liquid and semi-solid), a diverse set of environmental samples (Table 1) was used to discover new phages for a large phylogenetic range of bacterial hosts. Since semi-solid and liquid enrichments may capture different phages, both were used. Overall, 11 bacterial strains were tested against 9 different environmental samples (Supplementary Table S2). Phage plaques were observed in 31% (15/48) of the phage/host/sample combinations, and a subset of these phages was purified and sequenced (Supplementary Table S1). Using the HtPIP, we discovered 12 novel phages across 9 phylogenetically diverse bacterial host strains and recovered *P. putida* S12 phage MiCath from the same compost sample from which it was originally isolated (Jaryenneh et al., 2023). The new phages can be categorized into three taxonomic groupings: (1) maps to known species (Tolp), (2) maps to known genus (EW1 and Eliverwaste), (3) defines new genus and species (PLiverWaste, Gard, SweetSinh, Perlinasted, Prosser1, Prosser2, Maizie, Soil63, and Later). The large number of phages representing new phage genera highlights that the HtPIP can recover novel, highly diverse phages from non-model hosts.

**Figure 3:**
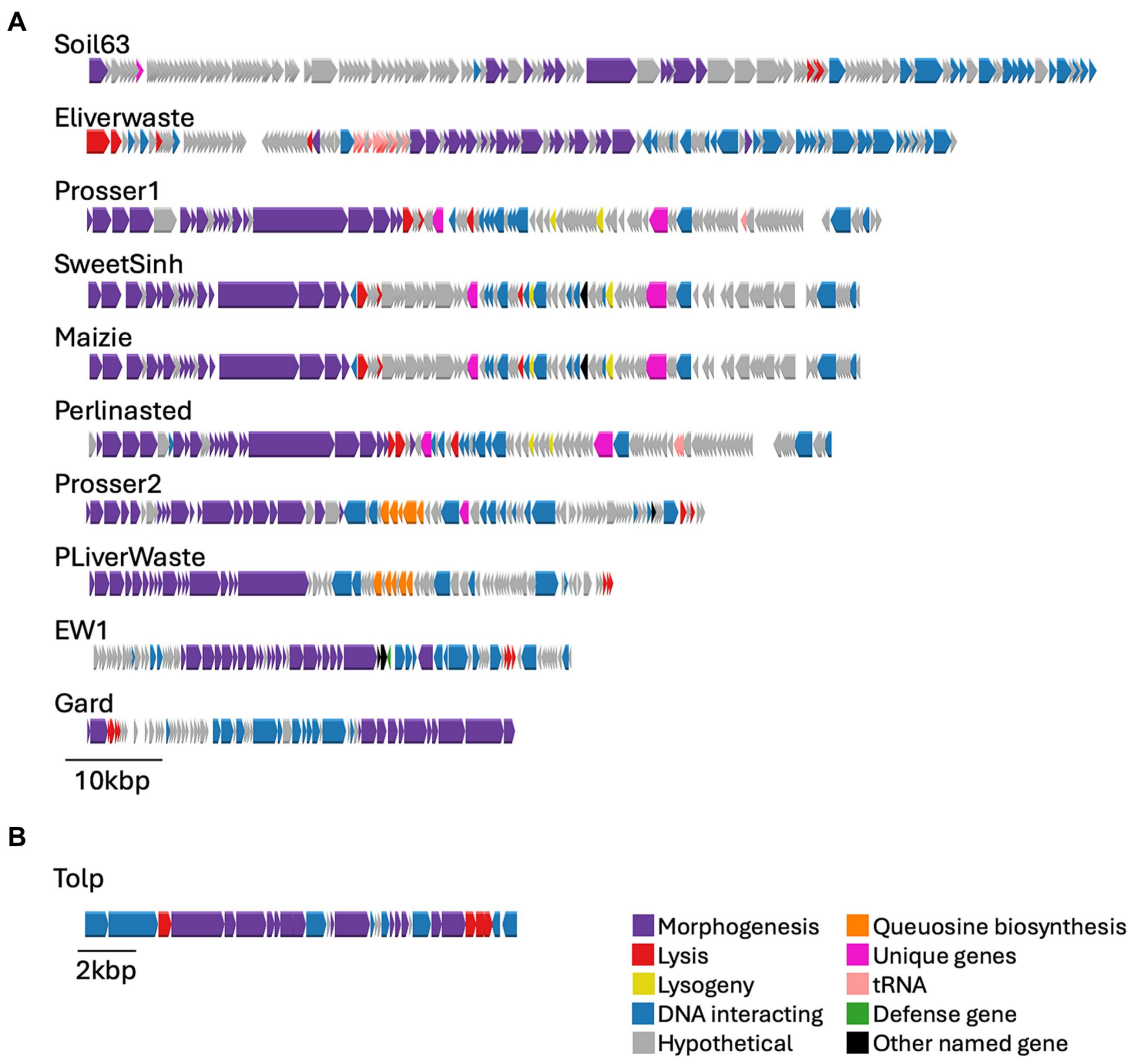
Genome maps of isolate phages discovered using HtPIP. Each phage genome is shown, and genes are colored according to function. **(A)** Shows all phages larger than 40 kbp ordered by size. **(B)** Shows phages smaller than 40 kbp. A size bar is below each series of maps.

The three phages that have close relatives are not identical to any previously isolated phage (genome statistics in Supplementary Table S1). Tolp is very similar to other *Tectiviridae* [97.74% ID over 100% genome to PRD1 (NC_001421.2)] and is a member of the *Alphatectivirus* PRD1 species. EW1 and Eliverwaste define novel species within their respective genera. EW1 is similar to T1, placing it in the *Tunavirus* genus, and the most closely related phage is vB_Henu5_2 [PQ362312.1] (90.56% ID over 95% of the genome). EW1 also carries the cor superinfection exclusion protein, which is used to prevent subsequent phage infection (Bucher and Czyż, 2024) (Figure 3A). Eliverwaste belongs to the genus *Felixounavirus* and is most similar to JLBYU32 [OK272490.1] (98.54% ID over 93% of the genome). Eliverwaste contains multiple tRNA genes and a ribonucleotide pathway (Figure 3A).

The remaining nine phages were novel and defined new genera and species of phages. The four with *Rhodococcus* hosts (Supplementary Table S1) could only be taxonomically determined to be in the *Caudoviricetes* class. Perlinasted was isolated on *R. qingshengii* S10 and resembles a previously isolated phage from our group, Perlina (PP782351.1; 97.43% identity across 98% of the genome) (Jaryenneh et al., 2024). Perlinasted contains 3tRNA genes (Figure 3A). SweetSinh and Maizie were both isolated on *R. rhodochrous* 372. They are 99.8% identical to each other, suggesting they are the same phage species, and are similar to *Rhodococcus* phage Reynauld (OR159659.1; 80.3% over 10% of the genome). Prosser1 was isolated on *Rhodococcus* sp. MSC1_016, an isolate from a novel soil consortium MSC-2 (McClure et al., 2022). Prosser1 shares little similarity with any phage at National Center for Biotechnology Information (NCBI); its closest relative is *Rhodococcus* phage Mbo2 (ON191531.1; 78.75% identity over 1% of the genome) and encodes a single tRNA gene. All *Rhodococcus* phages discovered contain a cas4 exonuclease and CobT-like cobalamin biosynthesis protein. All four phages are temperate phages encoding a tyrosine integrase (Figure 3A).

Two new Var*iovorax* phages were discovered: Gard, isolated on *Variovorax* sp. SCN, and Soil63, isolated on *Variovorax* sp. OAS795. Gard is taxonomically in the *Autographiviridae* family and most similar to *Variovorax* phage VAC51 (OX359471.1; 81.61% ID over 69% genome). Soil63 is a completely novel phage with only 228 bp homology found to any phage at NCBI [*Rhizobium* phage RHpl_I4 (MN988552.1)] and is found in the *Caudoviricetes* class. Soil63 has the largest genome (103.3kbp) of any phage discovered thus far using our HtPIP. Soil63 encodes a dCTP deaminase, which is typically classified as an auxiliary metabolic gene (Coutinho et al., 2023). Overall, due to the lack of sequence conservation, only 22.7% of genes (32/141) could be assigned a function (Figure 3A).

Although there have been many recent phages discovered for *P. putida* (Brauer et al., 2024; Jaryenneh et al., 2023), PLiverWaste is novel, sharing 73.81%ID over 2% of the genome with *Pseudomonas* phage Baskent P4_1 (PP992516.1) and in the *Caudoviricetes* class. PLiverWaste contains a queuosine biosynthesis pathway, suggesting the PLiverWaste DNA is protected from restriction endonucleases (Jaryenneh et al., 2023; Hutinet et al., 2019).

Prosser2 is a new *Sphingopyxis*, in the *Caudoviricetes* class, and has similarity to a previously isolated *Sphingomonas* phage vB StuS MMDA13 (NC_072503.1; 78.22%ID over 66% genome). Prosser2 has typical genes expected in a phage genome and a queuosine biosynthesis cassette, which has been shown to modify phage DNA to prevent restriction by the host (Jaryenneh et al., 2023; Hutinet et al., 2019).

**Figure 4:**
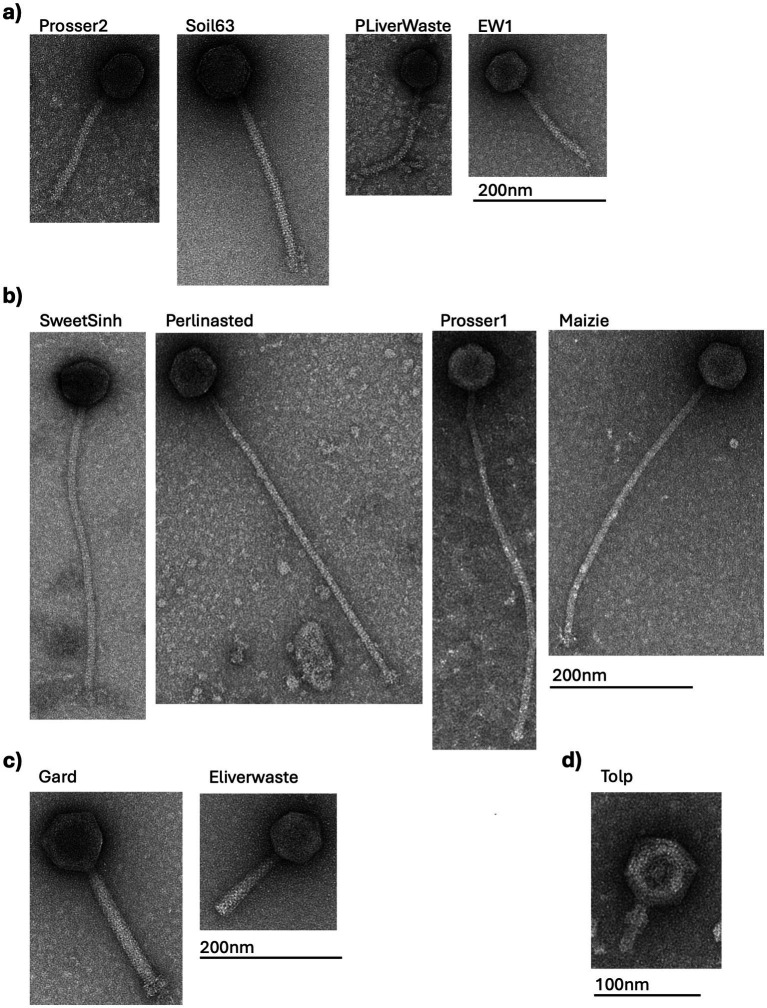
TEM images of all phages found using HtPIP. Here we show TEM images. We have grouped the phages by morphotype: **(A)** Phage with Siphoviridae morphology, **(B)** long-tailed Siphoviridae, **(C)** Myoviridae morphology, and **(D)** Tolp, a Tectiviridae. The scale bar for each grouping is shown below the image set.

To further characterize each of the eleven DNA phages above, TEM was used to determine the phage morphology (summarized in Supplementary Table S1). Prosser2, Soil63, and PLiverWaste all have typical siphoviridae morphology (Figure 4A). These phages all have a 1:3–4 head-to-tail ratio. The *Rhodococcus* phages (SweetSinh, Maizie, Perlinasted, and Prosser1) all have similar siphoviridae morphology with very long tails (400-460 nm), resulting in a head-to-tail ratio of 1:7 (Figure 4B). Gard and ELiverwaste were confirmed to have typical myoviridae morphology (Figure 4C), and Tolp was confirmed to have *Tectiviridae* morphology (Figure 4D).

### 3.3 HtPIP enables the discovery of the first *Levivirus* for *Microbacterium*

**Figure 5:**
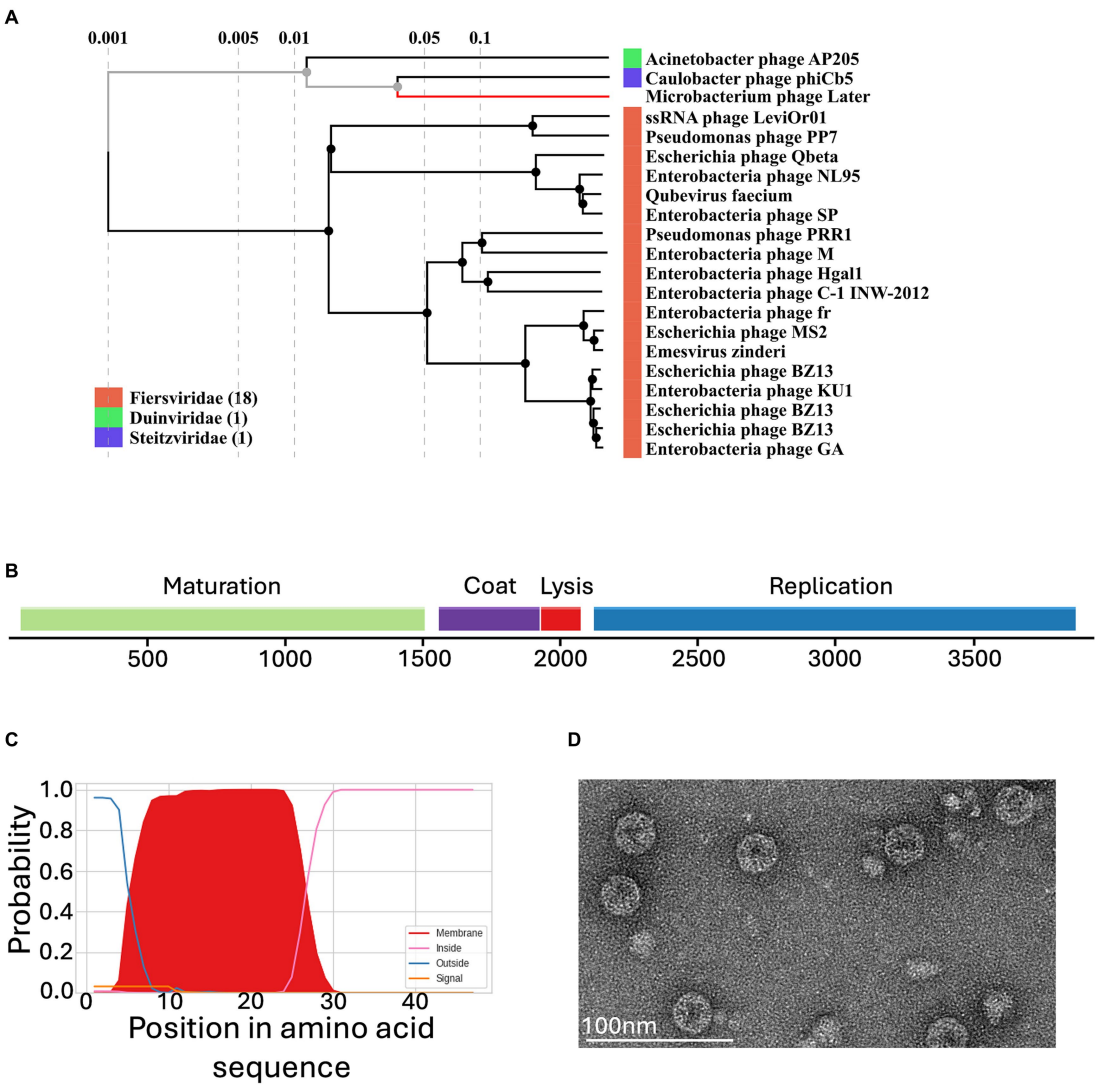
Isolation and characterization of *Microbacterium* phage Later. **(A)** VIP tree output for *Microbacterium* phage Later. Later appears to clade with the non-*Fiersviridae* in the VIP tree database. **(B)** The annotated genome of Later. Protein functions are listed above the gene. **(C)** DeepTMHMM posterior probabilities for ORF3 show one transmembrane region. **(D)** TEM of Later. The scale bar is shown in the lower left corner.

The twelfth novel phage discovered using HtPIP was a *Microbacterium* phage named “Later” isolated on *Microbacterium* sp. CM01. Later is an ssRNA virus in the *Leviviricetes* class with a 3,939 nt genome. The closest relative based on nucleotide sequence is a *Riboviria* sp. isolate parrot82 MAG (OQ424403.1; 79.11%ID over 56% of the genome). VIPtree (Nishimura et al., 2017) was used to characterize the phage genome further to find related phages based on protein similarity, which revealed homology with common *Leviviruses* such as MS2, Qbeta, PP7, and AP205 (Figure 5A). While these phages share protein similarity, phage Later cannot be placed into phage taxonomy further than the family *Steitzviridae* based on coat protein analysis (Callanan et al., 2021)*. Microbacterium* phage Later encodes proteins typically found in *Leviviridae* genomes (Ruokoranta et al., 2006; Chamakura et al., 2020), the maturation protein, coat protein, and replicase (Figure 5B). These results confirm HtPIP’s ability to discover RNA phages from environmental samples.

Since no *Leviviridae* have previously been described for *Actinobacteria,* and standard annotation tools could not identify the lysis protein (which is not unexpected for *Leviviricetes* due to low sequence homology (Ruokoranta et al., 2006; Chamakura et al., 2020)) previously established criteria were applied (Chamakura et al., 2020). There was only one predicted ORF that was annotated as a hypothetical protein (orf3). It was located between the coat protein and replication protein, a common location for *Leviviricetes* lysis proteins (Figure 5B). The predicted ORF was computationally analyzed for transmembrane domains (TMD), and a single TMD was predicted (Figure 5C), similar to other *Leviviridae* lysis proteins (Chamakura et al., 2020).

TEM confirmed that *Microbacterium* phage Later had *Leviviridae* morphology with small capsids, 28 nm × 25.4 nm, and no tail (Figure 5D), very similar to well-studied *Leviviricetes* that infect *Enterobacteria.* Together with the genomic analysis, the TEM findings confirm that *Microbacterium* phage Later is a member of *Leviviridae* and is the first reported to infect *Actinobacteria* species.

Phage Later infection was tested to understand host range, culture conditions, temperature impact, and the dependence on CaCl_2_. When tested against a panel of four *Rhodococcus* strains, three *Streptomyces* strains, and *E. coli* MFDPir, no plaques were observed, suggesting Later does not infect these species. Phage Later was spot-tested against *Microbacterium* sp. CM01 at 20 °C and 25 °C with the same efficiency of plating, but was unable to produce clearings at 30 °C and 37 °C (Supplementary Figure S1a). Furthermore, no evidence of bacterial lysis was observed at 20 °C in liquid culture experiments across the range of Phage Later concentrations (Supplementary Figure S1b). Finally, the addition of 1 mM CaCl_2_ had no impact on Phage Later infection in Microbacterium (Supplementary Figure 1a).

### 3.4 Phage diversity between HtPIP and traditional methods

Having demonstrated that HtPIP was compatible with a variety of environmental samples and bacteria, we were curious to see whether HtPIP captured different types of phages than traditional methods. We used *E. coli* MG1655 and a local wastewater influent sample for discovery, since total plate lysis was observed from our local wastewater samples on *E. coli* during the first two rounds of phage hunting with HtPIP (Supplementary Table S2). The viral lysate fraction was collected after initial enrichment from each method, extracted, and the metaviromic DNA sequenced and assembled into contigs >10kbp. Overall, 45 unique viral contigs were assembled between all sampling methods tested in this study.

**Figure 6:**
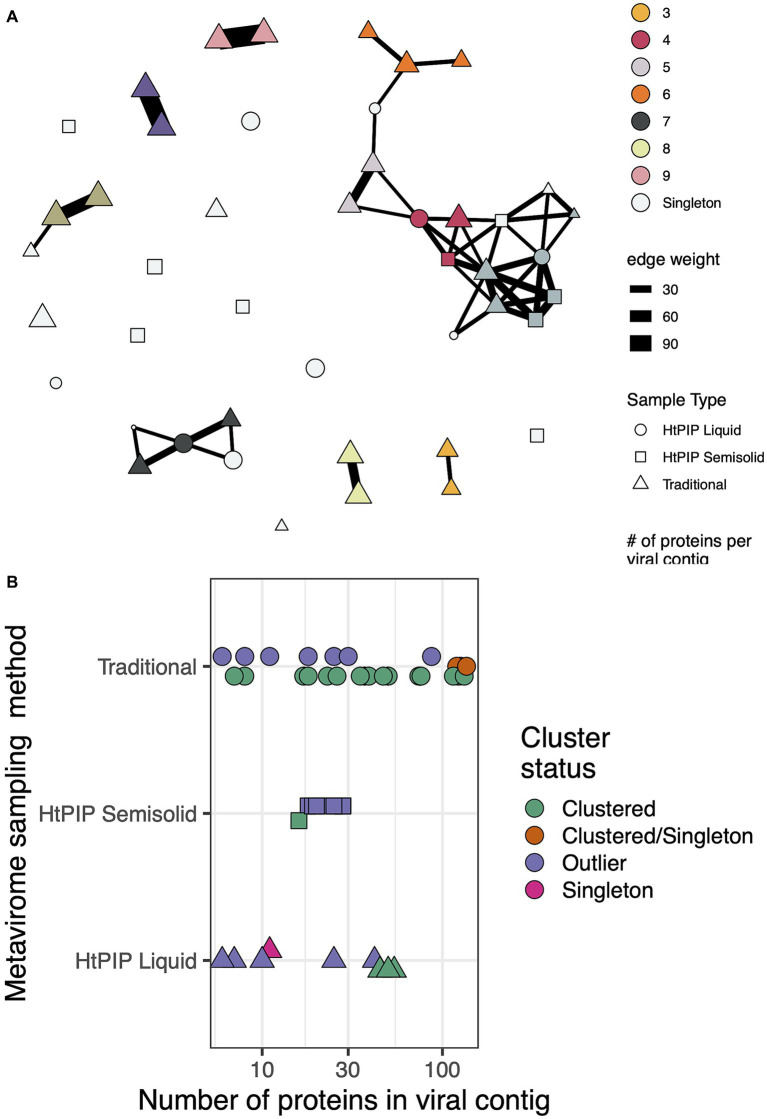
HtPIP, loaded with liquid and semisolid media, yields a higher proportion of novel viral contigs than traditional phage discovery. **(A)** Intra-study viral contig clustering via VContact2. “Traditional” represents a filtered wastewater influent sample mixed with overnight *Escherichia coli* diluted in LB and shaken for 3 days at 20 °C. The “HtPIP” samples originate from the HtPIP described in this paper. “Liquid” was when wells were filled with liquid LB, and “Solid” was when wells were filled with 0.5% LB agar. Clusters were determined by VContact2. “Singleton” samples were phage contigs with no discernible neighbor within the metaviromes assembled in this study. **(B)** Clustering phage contigs found in this study against the NCBI RefSeq database via VContact2. The *x*-axis denotes the number of proteins identified per contig, which typically indicates the size of the viral genome. Shapes labeled “Clustered” represent an assembled viral contig with one or more close neighbors among the RefSeq database, suggesting a shared genus-level classification with a previously sequenced phage. “Clustered/Singleton” contigs shared some DNA segments that clustered strongly and others that did not. “Outlier” contigs represent assembled viral contigs that clustered weakly with a known phage (based on edge weight), possibly representing a new genus. “Singleton” contigs did not cluster with any of thesequenced phages.

MVP (Coclet et al., 2024) was used to generate ORF predictions and the resultant protein sequences. Predicted proteins from the metaviromes were clustered with VContact2 (Bin Jang et al., 2019), which grouped the metaviromes into 10 unique viral clusters with at least two members among the three different sampling types, along with 18 singleton viral contigs (Figure 6A). Each cluster contained at least one member obtained by traditional methods (Figure 6A), indicating that the traditional method captured phages that were similar to each other. The HtPiP Liquid and HtPiP Semisolid methods each yielded 6 singleton phage contigs (12 in total) compared to the 5 captured by traditional methods, suggesting they captured a distinct array of phages compared to traditional techniques (Figure 6A).

To assess the novelty of the *E. coli* phages recovered using each phage discovery technique, the resulting viral contigs were clustered against the NCBI RefSeq repository. For this analysis, viral contigs were clustered with other known viral contigs and classified as “Clustered,” “Outlier,” or “Singleton.” Clustered samples have significant homology to known phages in RefSeq but may be a new strain or species. Outlier samples show weak similarity to known phages and may represent a new genus. Singleton contigs have no relatives at all and are the most unique category. Both HtPIP liquid and agar yielded a higher proportion of outlier contigs relative to clustered contigs (85 and 63%, respectively) than traditional methods (27%) (Figure 6B), suggesting that HtPIP captures a higher proportion of previously undiscovered phages than traditional methods.

## 4 Discussion

In this study, we have developed a novel high-throughput approach to discover phages for multiple bacterial hosts and have tested it on a variety of environmental samples. The optimal HtPIP setup recommended is a sealed plate top, inoculation at low host densities, and filtration by centrifugation for the highest yield of phages from environmental samples. We isolated 12 new phages for 9 different bacterial hosts. Many of these phages are novel in genomic and morphological terms compared with previously described phages and cannot be taxonomically classified beyond the family level. Within this set of novel phages, both DNA and RNA phages were isolated, along with multiple morphotypes and many newly predicted gene products. Additionally, we compared metaviromes obtained using our method vs. traditional methods and found that HtPIP captures a distinct set of phages compared to traditional methods (Figure 6A) and a higher proportion of previously undiscovered/outlier phages (Figure 6B). Importantly, the HtPIP technique saves time and requires simpler equipment for phage discovery, specifically during the initial filtering step of environmental samples; this step can involve up to 3 h of centrifuging, incubation, and filtering (Göller et al., 2020) and is not performed in our method.

The HtPIP is especially amenable to searching for phages across large-scale host collections, as the user can aliquot overnight cultures of a culture collection into multiple plates during the initial setup phase and incubate these replicate plates on top of several distinct environmental samples. The 96-well format is also compatible with automated lab equipment, such as liquid handlers and colony pickers, and downstream approaches to high-throughput phage isolation (Olsen et al., 2020). Although not explored in this study, this system could also be used in a time-series manner in environments where the phage ecology of the sample changes with time, as in soil samples (Wu et al., 2021; Van Goethem et al., 2019). Phage discovery from soil samples or other solid samples will especially benefit from using the HtPIP since filtering these sample types usually entails multiple centrifugation steps and prefiltration (Göller et al., 2020; Trubl et al., 2019). Furthermore, it is possible to adapt HtPIP to work *in situ* without risk of contaminating nearby soils or water since sealed plates are used.

While the HtPIP has enabled the discovery of many novel phages and provides a platform to accelerate phage discovery, it has limitations. One limitation is that some bacterial hosts seem to be more difficult to find phages for. We searched for *Burkholderia* phages in seven samples without success. Although not published in this article, we searched a similar number of samples from the same *Burkholderia* host using traditional methods and found no phages. This seems consistent with the few phages described in the literature for *Burkholderia*, so different techniques are likely needed to isolate phages for *Burkholderia* (Lauman and Dennis, 2021). The second limitation is downstream screening; in our current study, we still performed double-agar overlays to identify phage plaques after enrichment with the HtPIP. This step could be accelerated in the future by using automated lab equipment and workflows. Finally, our current HtPIP setup has a single reservoir, so only one environmental sample can be screened across up to 96 bacterial strains. While this significantly enhances the phage discovery process, many workflows will not require this many bacterial strains for screening. Custom plates can be developed to accommodate multiple reservoirs, potentially screening multiple environmental samples against fewer bacterial hosts, or these plates may be cut in half with a precision tool.

While phage discovery has accelerated in recent years (Cook et al., 2021; Shen et al., 2023) due to a renewed interest in phages for medical, health, and biotechnology applications (Santini, 2024), many bacteria still have few or no phage isolates (Cook et al., 2021; Roux and Mutalik, 2024). The HtPIP enables researchers to place up to 96 different bacteria into the plate with each sample, which increases the chance that a phage for a non-model bacterium may be found in an environmental sample. Discovery of phage isolates for non-model bacteria, especially those gaining interest as biomanufacturing chassis, provides valuable toolkits for these bacteria. These phages can be used to deliver gene cassettes to previously difficult-to-transform bacteria or to bacteria in microbial communities, or their gene products can be used to further enhance biotechnology applications (Lammens et al., 2020; Lemire et al., 2018).

One interesting discovery among our new phages was the extremely long tails of the *Rhodococcus* phages. Maizie, SweetSinh, Prosser1, and Perlinasted all have tails >400 nm, which are among some of the longest tails found on isolated phages that can be repeatedly infected. There is one phage described for *Rhodococcus equi*, ReqiDocB7, which also has a 489 nm tail (Summer et al., 2011). Very long flexible tails may be necessary to go through the thick cell wall of *Rhodococcus* (Piselli et al., 2022).

The HtPIP facilitated the discovery of new *Tectiviridae* (Figure 4), which are difficult to isolate using traditional methods. *Tectiviridae* are known-to-infect *Pseudomonas* species, which carry plasmids for pili formation (Grahn et al., 2006; Saren et al., 2005). We confirm here our new phage Tolp can infect *P. putida* mt-2, which harbors the pWW0 plasmid that contains genes for conjugative transfer and toluene and xylene degradation (Burlage et al., 1989; Greated et al., 2002), but not *P. putida KT2440*, which lacks this plasmid (Supplementary Table S2).

The most taxonomically novel phage found using the HtPIP was the RNA phage Later; it was confirmed through genomic sequencing and TEM to be a *Leviviricete* infecting a soil *Microbacterium* isolate (Figure 5). This demonstrates the ability of HtPIP to capture both DNA and RNA viruses, and is the first RNA phage isolated outside of proteobacteria (Krishnamurthy et al., 2016). The only other RNA phage described outside of proteobacteria is *Streptomyces* phage ϕ0, a *Cystovirdae* phage, uncovered in a pure-culture transcriptome experiment from *Streptomyces avermitilis* (Callanan et al., 2021; Krishnamurthy et al., 2016). Although *Leviviridae* isolates have been described for many Gram-negative hosts, including *Enterobacteria*, *Pseudomonas*, *Acinetobacter*, and *Caulobacter*, to date, there has been no *Leviviridae* isolated for *Actinobacteria* (Callanan et al., 2021; Kazaks et al., 2011; Fiers et al., 1976; Klovins et al., 2002; Kim et al., 2018) or any Gram-positive host to our knowledge. Many outstanding questions remain to be explored for Later, especially regarding phage-host interactions required for entry. All *Leviviridae* described use Gram-negative bacterial pili to begin the infection process (Hör, 2025). While our *Microbacterium* isolate has an annotated Type IV pilus assembly operon, it is unknown whether the mechanism of phage entry differs in Gram-positive bacteria. The temperature during host growth, as well as the substrate (liquid/planktonic vs. semisolid/biofilm), affects host cell lysis by phage Later (Supplementary Figure S1). These factors may be influencing host pili activity or other host processes. It is also possible that quorum signaling agents or antibiotics produced by the native bacterial community growing in the reservoir beneath the semipermeable plate may promote the expression of surface structures similar to pili (Lu et al., 2017) that are critical for *Leviviridae* attachment and subsequent infection. These and possibly other unknown chemicals exchanged between the growing native bacterial community and the monoculture hosts grown on the other side of the membrane warrant further research to understand better how the microbiome community affects phage infection dynamics.

## 5 Conclusion

Here we present a novel phage discovery platform (HtPIP), which grows bacterial hosts in proximity to environmental samples such as soil and sewage influent, separated by a 0.2-μM membrane. We optimized a procedure to capture the majority of the phages using model bacterial hosts. We tested the HtPIP across a variety of environmental samples to discover phages for multiple hosts at once. Using this method, DNA and RNA phages infecting a diverse range of hosts were isolated, including both Gram-negative and Gram-positive bacteria. A higher proportion of unique phage contigs was assembled from HtPIP metaviromes compared to traditional phage discovery metaviromes. We envision researchers using HtPIP can efficiently discover, from a single environmental sample, diverse and novel phages for up to 96 bacterial hosts.

## Data Availability

The metaviromics datasets generated in this study can be found in the sequence read archive (SRA) under BioProject PRJNA1415247, Biosamples SAMN54927485-SAMN54927489 and SAMN54985761. All phage genomes were deposited into NCBI GenBank, accession numbers are listed in Supplementary Table 1. Microbacterium sp. CM01 sequence was deposited at NCBI GenBank under Bioproject PRJNA144606 and BioSample SAMN56810849.
