# Supplementary material for: HtPIP: High-throughput phage isolation platform increases diversity and reduces isolation time using multiple bacteria
Source: Front Microbiol. 2026 Jun 18;17:1845440. doi: 10.3389/fmicb.2026.1845440 (PMC13351103; doi:10.3389/fmicb.2026.1845440)
Supplement: Supplementary file 1 [file Data_Sheet_1.PDF]

Supplementary Materials

Supplemental Table 1. Collection information, genotypic, and phenotypic information about all isolated phages.

	**Wastewater Influent 1**	**Compost - backyard**	**Wastewater influent 2**	**Commercial Topsoil**	**Tomato rhizosphere**	**Wastewater influent 3**	**Compost - community center**	**Agricultural soil**
E. coli MG1655								
P. putida S12								
P. putida mt-2								
*B. cenocepacia K*56-2								
Microbacterium sp. MSC1_018								
R. rhodochrous 372								
R. qingshengii S10								
Variovorax sp. SCN								
Variovorax sp. OAS795								
S. fribergensis MSC1_008								
Rhodococcus sp. MSC1_016								

Supplementary Table 2. Environmental Sample and Bacterial Host Matrix. Light blue boxes denote plaques isolated from enrichments; dark blue denotes web of phages were isolated. White denotes no phages were found. Grey boxes denote not tested. The total combinations of strains and samples tested was 54.

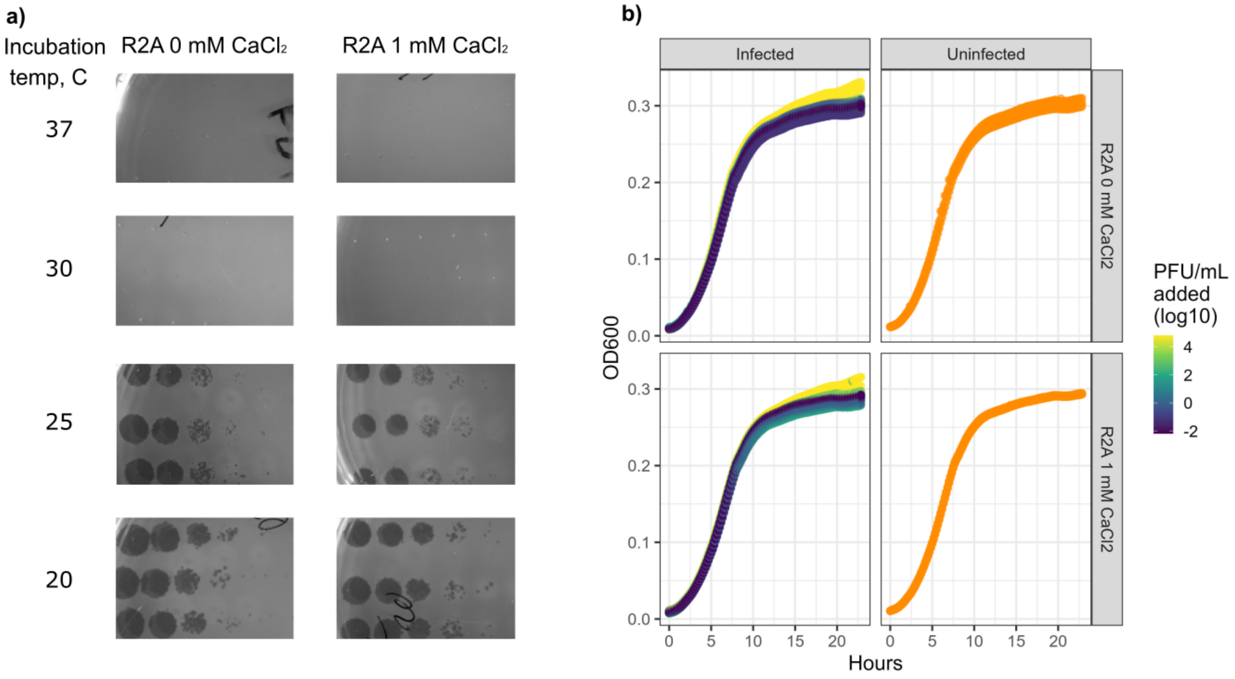

Supplementary Figure 1. Characterization of *Microbacterium* phage Later infection dynamics. a) Efficiency of plating of phage Later on *Microbacterium* CM01 R2A media with and without supplemental $CaCl_2$ (1mM) added at a range of temperatures. Replicate plates were incubated for 24 hours at indicated temperature. **b)** Liquid infection assays for Phage Later with *Microbacterium* CM01 with and without supplemental $CaCl_2$ (1mM) at 20C. Liquid infection at various viral concentrations shows no apparent growth defect compared to uninfected *Microbacterium* sp. CM01, regardless of calcium supplementation.
